# Spatiotemporal characterisation of information coding in the multiple demand network

**DOI:** 10.1162/IMAG.a.1171

**Published:** 2026-03-20

**Authors:** Hamid Karimi-Rouzbahani, Anina N. Rich, Alexandra Woolgar

**Affiliations:** Medical Research Council Cognition and Brain Sciences Unit, University of Cambridge, Cambridge, United Kingdom; Queensland Brain Institute, University of Queensland, Brisbane, Australia; Macquarie University Performance & Expertise Research Centre and School of Psychological Sciences, Macquarie University, Sydney, Australia; Department of Psychology, University of Cambridge, Cambridge, United Kingdom

**Keywords:** multiple-demand network, MEG, fMRI, decoding, temporal dynamics

## Abstract

The multiple-demand network (MDN), a set of highly interconnected, domain-general regions active across a wide variety of cognitively demanding tasks, is thought to support cognitive functions by integrating distinct types of information depending on the task. However, the spatiotemporal characteristics with which each node in the MDN encodes information remains unclear. We collected functional magnetic resonance imaging (fMRI) and magnetoencephalography (MEG) data from separate participants performing a complex visual stimulus–response mapping task. We used multivariate pattern analysis (MVPA) to decode various task-related types of information—stimulus details, motor responses, and mapping rules—in both the MDN and visual areas. We used model-based MEG–fMRI fusion to compare the high temporal resolution data from MEG with high spatial resolution data from fMRI, extracting commonalities that reflect both the time course and location with which these different task features were represented. Early on, visual regions encoded information about the visual hemifield of the stimulus, while later, the MDN encoded the fine-grained details of the stimuli within the same hemifield and the task rules. We observed distinct temporal profiles of information coding for the cingulo-opercular versus frontoparietal sub-networks of the MDN. This study offers insights into the dynamic information processing of the MDN and provides information-coding-based support for at least two sub-networks within the multiple-demand network.

## 1 Introduction

Humans are able to engage in intelligent goal-directed behaviour across different scenarios. To achieve this, the brain must combine information from multiple systems, from the sensory input of multiple modalities, through to memory, attention, and motor processes. These systems are distributed across the brain, and a key question in understanding intelligent behaviour is how such information is integrated and coordinated.

A prominent candidate for this high-level executive function is a network of regions known as the multiple demand network (MDN; Duncan et al., 2020), also called the frontoparietal control network (e.g., Marek & Dosenbach, 2018), or cognitive control network/networks (e.g., Cole & Schneider, 2007). This network consists of regions in the intraparietal sulcus (IPS), anterior insula/frontal operculum (AI/FO), inferior frontal sulcus (IFS), and dorsal anterior cingulate cortex (ACC)/pre-supplementary motor area (pre-SMA) and overlaps considerably with the frontoparietal resting-state network (Assem et al., 2020). It is active in many cognitive tasks (Assem et al., 2020; Fedorenko et al., 2013) and is widely thought to have a key role in supporting intelligent flexible behaviour (Colom et al., 2010; Duncan, 2010; Jung & Haier, 2007). For example, the extent of damage to these regions (and not nearby language regions) linearly predicts deficits in fluid intelligence (Woolgar et al., 2010, 2018), the ability to think abstractly and solve novel tasks/problems that is predictive of a wide range of cognitive abilities (Baltes et al., 1999).

In line with its putative role in supporting a wide range of goal-directed behaviours, the MDN shows remarkable flexibility in adaptively coding information across a wide range of tasks (Zheng et al., 2024). Univariate functional magnetic resonance imaging (fMRI) studies routinely show activation in this network across cognitive tasks, including those involving memory, maths, conflict detection, visual discrimination, and more (e.g., Assem et al., 2020; Fedorenko et al., 2013; Stiers et al., 2010), suggesting its involvement in cognitively challenging tasks regardless of specific content (Dosenbach et al., 2006; Duncan & Owen, 2000). Multivariate fMRI studies have demonstrated that the MDN differentiates a range of different types of information (including stimuli, rules, and responses) across tasks (Crittenden et al., 2016; Shashidhara et al., 2024; Woolgar et al., 2016). Moreover, within tasks, MDN responses are strongly shaped by task relevance (Zheng et al., 2024) and difficulty (Stiers et al., 2010; Woolgar et al., 2011a, 2015b). For example, representations of identical stimuli were enhanced in the MDN when attention was directed to objects (Woolgar et al., 2015b), object features (Jackson & Woolgar, 2018; Moerel et al., 2024; Wisniewski et al., 2023), or both (Dermody et al., 2025). Representations across the MDN were also enhanced for difficult versus easy stimuli (Woolgar et al., 2011a, 2015b) and rules (Woolgar et al., 2015a). By combining transcranial magnetic stimulation (TMS) and fMRI, it has further been demonstrated that the right dorsolateral prefrontal cortex node (dlPFC) of the MDN plays a causal role in the dominance of task-relevant information in the system specifically by upregulating task-relevant codes (Jackson et al., 2021).

There is also evidence that connectivity and information coding in the MDN can predict behavioural performance. For example, in an auditory detection task, pre-stimulus connectivity between auditory sensory areas and cingulo-opercular nodes of the MDN (i.e., ACC/pre-SMA and AI/FO) correlated with the accuracy of behavioural responses (Sadaghiani et al., 2015). In a stimulus–response mapping study, where different rules determined the mapping between stimuli and response buttons, the information held by the MDN correlated with behavioural responses, such that when the response was incorrect, the MDN also represented incorrect information (Woolgar et al., 2019; see also Robinson et al., 2022). These results suggest an important role for the MDN in processing task-related information and supporting behaviour.

One proposal is that the MDN could underpin flexible goal-directed behaviour by integrating information arising in distinct and distant cognitive systems of the brain (Cole et al., 2013; Duncan et al., 2020; Jung & Haier, 2007; Zheng et al., 2024). This requires functional connections between the areas involved. Indeed, in addition to co-activation and flexible encoding of information across tasks, there is also evidence from resting-state fMRI studies that, compared with non-MD areas, the nodes of the MDN are highly connected (Assem et al., 2020; Cole et al., 2010; Power et al., 2013). They follow a small-world network structure (Bullmore & Sporns, 2009), which can facilitate access to distant parts of the brain through relatively short pathways compared with a regular network where information needs to pass many nodes to move across the brain. Such connections provide a potential mechanism by which the MDN could bring together different sources of information in arbitrary ways (Cole et al., 2013; Duncan et al., 2020; Higo et al., 2011). Indeed, information coding in individual MD regions can be predicted from the information held in connected regions (Schultz et al., 2022).

A parallel line of research into the temporal dynamics of cognitive control has shown that information coding in the brain can change rapidly in the time domain (Zheng et al., 2024). For example, using time-resolved neuroimaging (magnetoencephalography, MEG, electroencephalography, EEG), information at the focus of selective attention has been repeatedly shown to be coded more strongly and in a more sustained manner than equivalent irrelevant information, from around 200 ms after stimulus onset (Barnes et al., 2022; Battistoni et al., 2020; Goddard et al., 2022; Moerel et al., 2022, 2024; Noah et al., 2023). Again, frontoparietal cortex is implicated. For example, information coding in frontal cortex appears to shape information coding in visual cortices at later time points (Goddard et al., 2022), while causal modulation of parietal cortex influences brain-wide coding of spatial attention in a manner that predicts behaviour (Lu et al., 2025). However, the relatively poor temporal resolution of human fMRI has made it difficult to examine whether these findings pertain to the MD system specifically.

Thus, on the one hand, the field of fMRI strongly implicates specific frontoparietal brain regions, the MD system, in integrating diverse types of information to solve challenging tasks. On the other, time-resolved neuroimaging shows that the brain is capable of rapid dynamic re-configuration according to task demands. However, the relationship between the two observations has largely been implicit rather than formalised. Here, we attempted to bridge these observations by formally comparing fMRI and MEG data collected on a single, challenging, stimulus–response task, and drew on the spatiotemporal richness of fused MEG–fMRI data to study the temporal dynamics of information coding across the nodes of the MDN. We collected fMRI data while participants performed a paradigm that we had used previously in MEG (Robinson et al., 2022) and characterise the dynamic representation of stimuli, rules and button-press responses in each MDN node. Following previous observations that MD regions show particular focus on representation of more challenging aspects of visual tasks (e.g., Woolgar et al., 2011a, 2015b), we additionally separated representation of the more challenging (fine-grained) aspects of visual stimuli (discrimination of nearby locations within hemifield) from more fundamental visual signals (separation of the two hemifields).

This specific stimulus–response mapping task was chosen because it incorporates the main components of a cognitive task (stimulus processing, rule application, and response selection) while remaining simple enough to allow for clear component assignment. Previous studies have shown that the MDN is robustly engaged by these task types, particularly when encoding task-relevant information, attended features, and difficult or arbitrary rules (e.g., Woolgar et al., 2011a, 2015a, 2016). We chose the task to specifically allow us to investigate whether distinct aspects of task information modulate the involvement of the MDN sub-networks.

To investigate the temporal specialisation of the MDN, we adopted a hypothesis-driven approach using ROIs defined by extensive prior data-driven mapping (Assem et al., 2020; Duncan & Owen, 2000; Fedorenko et al., 2013) and studied in the context of this and similar tasks (e.g., Woolgar et al., 2011b, 2016). By fusing the spatial precision of fMRI with the superior temporal resolution of MEG sensor-space data, we resolved fine-grained dynamics within specific MDN nodes while avoiding the MEG inverse problem (Hämäläinen & Ilmoniemi, 1994). This focused strategy allowed us to uncover subtle temporal differences between MDN sub-networks—such as the frontoparietal and cingulo-opercular systems—that are inaccessible through fMRI alone. Ultimately, this approach maximised the statistical power necessary to provide novel insights into the rapid, within-trial information processing of the human MDN.

We found that task-related information was encoded with different dynamics in different areas. There was earlier and stronger information coding in early visual cortex (EVC) than MDN, and there was a discriminable coding profile between some MDN nodes, potentially aligning with proposed cingulo-opercular and the frontoparietal sub-networks, which have shown distinct temporal profiles (Crittenden et al., 2016; Dosenbach et al., 2006). This detailed spatiotemporal characterisation provides insights into how information is encoded within and beyond the MDN in support of flexible rule-based behaviour.

## 2 Methods

We collected fMRI data using the same paradigm for which we had previously collected MEG data (Robinson et al., 2022), with new participants. The paradigm is designed to investigate the encoding of stimulus, rule, and response in a stimulus–response mapping task and has shown a strong link between behavioural performance and neural coding of information (Woolgar et al., 2019). Specifically, at around the time of response, the coding of stimulus representations predicted whether participants would give a correct or incorrect response (Robinson et al., 2022). We combined this MEG dataset with our new fMRI dataset to explore the role of the MDN in encoding and integrating task information for flexible behaviour.

### 2.1 Participants

In total, 30 (14 female, 16 male; age: mean = 24.4 years, sd = 2.1) participants were recruited from Macquarie University volunteer panel for the fMRI study. No fMRI participant had participated in the previous MEG study (see Robinson et al., 2022, for full methodological details of the MEG study). Participants were right-handed and had normal or corrected-to-normal (through contact lenses) vision. Participants gave written informed consent for both a behavioural training of 1 hour prior to scanning (reimbursed AU$15) and the 2-hour fMRI experiment (including setup; reimbursed AU$40). The study was approved by the ethics committee of Macquarie University.

### 2.2 Task design

**Fig. 1.**
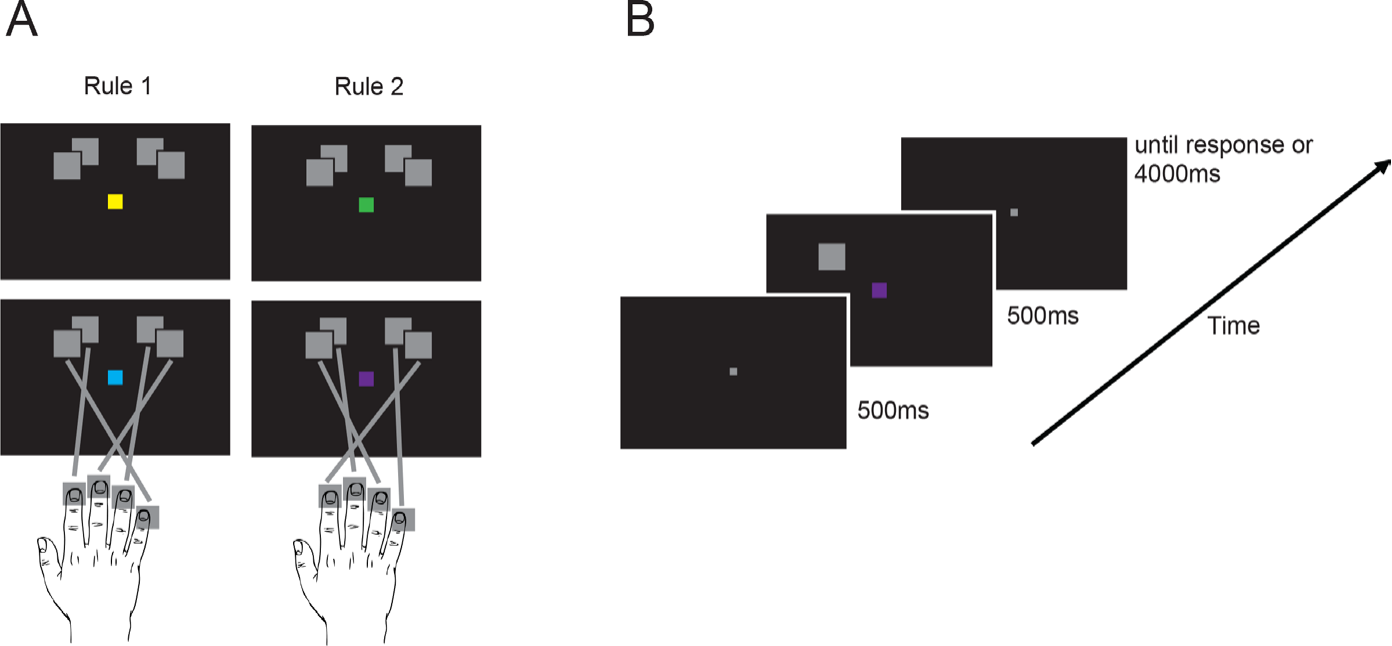
Experimental paradigm and the definition of different types of information (figure redrawn from Robinson et al., 2022). (A) Experiment was a stimulus–response mapping task, where participants had to press the button that corresponded to each stimulus. Participants were trained to perform two distinct stimulus–response mappings (rules) which were indicated by the colour of fixation square (two per rule). We defined the across-hemifield stimulus information as the distinction between the stimuli presented on the left versus right hemifield (collapsed across inner and outer positions) and the within-hemifield stimulus information as the distinction between the stimuli presented in the inner versus outer positions in the same visual hemifield (collapsed across left and right). Rule information refers to the distinction between the two rules and response information refers to the distinction between responses made using inner versus outer fingers (collapsed across stimuli and rules). (B) Participants were cued about which rule to apply on a trial-by-trial basis. Each trial started with a fixation cross, followed by a stimulus-rule cue display and then the response screen.

We collected fMRI data while participants performed a visual stimulus–response mapping task (Fig. 1; Robinson et al., 2022) designed to tease apart stimulus, cue, rule, and response encoding in the brain. The task involved pressing of one of four buttons according to the position of a visual stimulus and one of two memorised stimulus–response mapping rules (Fig. 1A). Each trial started with the presentation of a grey fixation square for 500 ms followed by the stimulus for another 500 ms after which the participants could provide a response (Fig. 1B). The response screen lasted for 4,000 ms or until the response was made, whichever happened first.

In the initial behavioural training session, participants had to learn two sets of mappings between stimuli and the buttons (rules), as indicated by the colour cue on the centre of the screen. Each rule was indicated by two cue colours (giving a total of four possible cue colours: two indicated that Rule 1 was to be applied, and the other two indicated that Rule 2 was to be applied) to allow us to see the effect of cue colour on rule processing, and to avoid the classifiers using cue colour to distinguish the categories. Specific cue colour–rule associations were randomised and counterbalanced across participants. Participants responded using the four fingers of their right hand.

Stimuli were presented and responses collected using MATLAB Psychtoolbox (Brainard & Vision, 1997). Our training session was identical to the one used in the MEG study (Robinson et al., 2022): the task started with easier versions, in which stimuli were presented in non-overlapping positions in earlier blocks, to the final version, with overlapping stimuli, like the version used in the MRI scanner. Rules were trained one by one in the first 2 blocks, each block containing 32 trials. Participants needed to reach 60% accuracy to continue to the subsequent block, otherwise the block would repeat until they reached the threshold, or they were excluded and replaced if they did not reach it after 5 consecutive blocks. Participants did an average of 8.61 (sd = 2.46) training blocks to finish the training session successfully.

### 2.3 Apparatus

FMRI scans were acquired using a Siemens 3 T Verio scanner with 32-channel head coil, at the Macquarie Medical Imaging centre, Macquarie University Hospital, Sydney, Australia. We used an interleaved ascending T2*-weighted echo planar imaging (EPI) acquisition sequence with the following parameters: repetition time (TR), 2,000 ms; echo time (TE), 30 ms; 36 slices of 3.0 mm slice thickness with a 0.366 mm interslice gap; in-plane resolution, 3.0 × 3.0 mm; field of view, 126 mm. We also acquired T1-weighted MPRAGE structural images for all participants (non-selective inversion recovery, resolution 1.0 × 1.0 × 1.0 mm). The session began with the collection of a T1 image which lasted for about 5 minutes followed by EPI acquisition for the blocks of experimental trials.

Each block started with the presentation of a rule screen which showed the four cue conditions, two per rule, for 10 seconds, followed by the experimental trials. We acquired six scanning runs, each consisting of two blocks. Each run lasted for ~8 minutes after which the data were saved and scanning restarted. Within each block, there were 80 trials presented in pseudorandom order such that all stimulus configurations (4 cue colours × 4 stimuli) appeared 5 times with equal probability within each block. Stimuli were presented on an LCD screen at the back of the scanner, and participants could see the LCD through a head-coil mounted mirror.

### 2.4 Pre-processing

We pre-processed the fMRI data using SPM 12 (Wellcome Department of Imaging Neuroscience, www.fil.ion.ucl.ac.uk/spm) in Matlab 2018a. DICOM data were converted to NIFTII format. Functional images were slice-time corrected and realigned to the first functional scan in the run. They were then smoothed slightly (4 mm FWHM Gaussian kernel) to increase the signal-to-noise ratio as in previous work (Jackson et al., 2017; Woolgar et al., 2015b). Structural images were co-registered to the mean functional images and normalised to the MNI152 space (McConnell Brain Imaging Centre, Montreal Neurological Institute) to derive normalisation parameters that we later used to define Regions of Interest for individual participants.

The MEG pre-processing procedure is explained in the MEG study in detail (Robinson et al., 2022). Briefly, the signals were band-passed (0.03–200 Hz), notch-filtered (50 Hz), and down-sampled to 200 Hz with no additional pre-processing steps. Data were cut into *stimulus-aligned* epochs from -200 before to 2,500 ms after stimulus onset and *response-aligned* epochs from -2,500 to 500 ms after response. Signals are analysed in the 160-channel sensor space.

### 2.5 Regions of interest (ROIs)

Template space MDN ROIs were taken from our previous work (Woolgar et al., 2015b) where they were defined based on their activity in a wide range of cognitive tasks (Duncan, 2010). These consist of left and right inferior frontal sulcus (IFS; centre of mass +/−38 26 24, volume 17,000 mm^3^); left and right anterior insula/frontal operculum (AI/FO; +/−35 19 3, 3,000 mm^3^); left and right intraparietal sulcus (IPS; +/−35 −58 41, 7,000 mm^3^), and the dorsal anterior cingulate area (ACC; +/−0 23 39, 21,000 mm^3^).

The visual areas were obtained from the Brodmann template provided with MRIcro (Rorden & Brett, 2000): Brodmann area 17/18 (EVC; −13 −81 3, 16 −79 3, 54,000 mm^3^). The lateral occipital complex (LOC; +/− 40 − 70 – 9, 41,800 mm^3^) was defined from a prior review of 8 imaging studies reporting greater activation for objects compared with scrambled shapes or textures (Thompson & Duncan, 2009). ROIs were defined based on a subjects-averaged localiser data from a previous study where we contrasted the areas which responded more strongly to pictures of natural/man-made objects than to scrambled versions of the same objects (Jackson et al., 2017). Our LOC ROIs fell very close to anatomical LOC coordinates from previous studies (Grill-Spector et al., 1999, 2000). All ROIs were defined and analysed in each hemisphere separately, but decoding results were averaged over hemispheres for inference.

### 2.6 First-level model

**Fig. 2.**
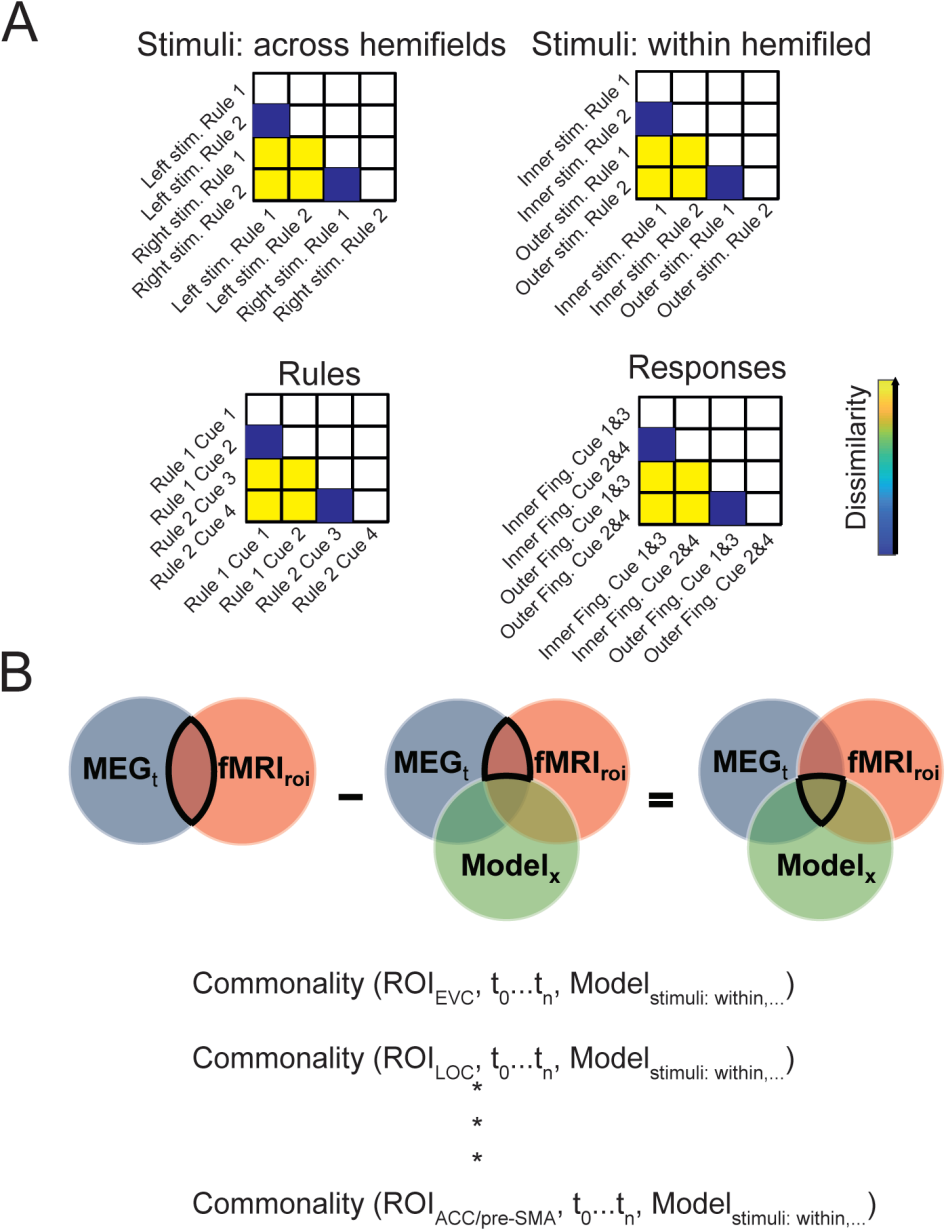
Commonalities between fMRI, MEG, and models. (A) Four distinct neural Representational Dissimilarity Matrices (RDMs) were constructed for each fMRI ROI and each MEG time point by different arrangements of stimuli, rules, cues, and responses, which allowed us to evaluate the commonality between fMRI and MEG representations and target models (here we are showing the models rather than neural RDMs). Note that the rows and columns of these matrices are different for each type of information (see Methods). Model RDMs had identical arrangements of conditions to the neural RDMs but only included binary values (1 for high and 0.5 for low dissimilarity). (B) RDMs from every fMRI Region of Interest (ROI), every MEG time point, and every model were entered into the commonality index equation (equation (1), see Methods), resulting in a commonality trace for every ROI and time point. The commonality index for every ROI and time point (highlighted on the right Venn diagram) is calculated as the difference between MEG_t_.fMRI_roi_ correlation (highlighted on the left Venn diagram) and MEG_t_.fMRI_roi_ correlation while the Model_x_ is partialled out (highlighted on the middle Venn diagram).

To estimate the activation patterns (beta values) for each condition and each area, we used a General Linear Model (GLM), convolving event regressors with the duration of the trial reaction time (Grinband et al., 2008) with the first-order haemodynamic response of SPM. For MVPA, we used a total of eight regressors representing *across*-stimulus information (the two stimuli on the left vs. the two on the right side of space), *within*-stimulus information (the two stimuli located in the inner vs. outer peripheral positions relative to the central fixation square), *rule* (rule 1 vs. rule 2), and *response* (inner vs. outer buttons). Each trial contributed to the estimation of four of these regressors. For Representational Similarity Analysis (RSA), we ran four separate sets of GLMs on the data to maximise the power for the desired information to avoid subtle information (e.g., rule) being dominated by stronger information (e.g., *across*-hemifield stimulus)^1^ and to have enough regressors for RSA (see Fig. 2A). Specifically, to construct a 4 × 4 representational dissimilarity matrix (RDM) for *across*-stimulus information, we included two regressors for the *across*-stimulus information (left vs. right) and two regressors for the rule information (rule 1 vs. rule 2). To construct the 4 × 4 RDM for the *within*-stimulus information, we included two regressors for the *within*-stimulus information (inner vs. outer) and two regressors for the rule information (rule 1 vs. rule 2). To construct the 4 × 4 RDM for rule information, we included four regressors for the rule information (all four cue colours). Finally, to construct the 4 × 4 RDM for response information, we included two regressors for the response information (inner vs. outer buttons) and two regressors for the cue colours information collapsing across rules to avoid their effect (one regressor for each of two colours from different rules). For the RSA GLMs, each trial contributed to the estimation of two regressors (e.g., *across*-hemifield stimulus information and rule). In all GLMs, movement parameters, block means, and run means were also included as covariates of no interest, and trials were modelled as epochs lasting from stimulus onset until response (Grinband et al., 2008). Error trials were excluded from the analysis. We estimated the regressors for each of the 12 blocks of each participant obtaining 12 beta values which we used in MVPA.

### 2.7 Multivariate pattern analysis (MVPA)

We used standard multivariate pattern decoding to quantify the level of different types of information consisting of *across* and *within*-stimulus, rule, and response information, as defined above, in fMRI and MEG.

In fMRI, we used a leave-one-block-out cross-validation approach for decoding using a linear SVM classifier as implemented in MATLAB version: R2020b. This way, we trained the classifier on all-minus-one blocks and tested the classifier on the remaining one block and repeated the procedure until all blocks were used in testing once. The decoding accuracy for a single participant was calculated by averaging the decoding results across cross-validation runs. The number of voxels within each ROI determined the dimension of the data entered the classifier. The classification was performed for each ROI in each hemisphere separately and then averaged across the two hemispheres and reported.

In MEG, we used a time-resolved decoding procedure (Robinson et al., 2022), where we repeated the decoding analysis on every time point along the trial, once using the data aligned to the stimulus onset time (*stimulus-aligned* analysis) and once with the data aligned to the response time of each trial (*response-aligned* analysis). We included all 160 MEG channels in decoding. The two different alignments provide insights about the temporal profile of information encoding affected by stimulus onset and those aligned to the behavioural outputs. We used a 10-fold cross-validation procedure, with 9 folds of the data used for training the classifier and the left-out fold used for testing it and repeating the procedure 10 times until all folds are used once in testing. The decoding accuracy for a single participant was calculated by averaging the decoding results across these cross-validation runs.

### 2.8 RSA-based fMRI–MEG fusion

We used RSA to provide a common platform for comparing the representation of information across ROIs in fMRI and across time in MEG (Kriegeskorte et al., 2008). For each participant (different in fMRI and MEG), ROI in fMRI, or time point in MEG, we constructed four distinct neural representational dissimilarity matrices (RDMs) by decoding different combinations of stimuli, cues, rules, and responses (Fig. 2A). These four RDMs were designed to capture *across-* and *within*-stimulus, rule, and response information. Each cell in the RDM reflects the decoding accuracy of the two conditions of the column and row of the matrix. For example, the decoding value at the intersection of column “Right stim. Rule 1” and row “Left stim. Rule 1” contains the decoding of right stimuli in rule 1 and left stimuli in rule 1, therefore, reflecting information (dissimilarity) about stimulus side irrespective of other variables such as rule. We chose decoding-based RDMs for two reasons: 1. Functional Relevance (Linear Readout): Decoding accuracy, particularly from a linear classifier, provides a direct measure of the linear separability of the neural representations. This metric is argued to be a more ecologically valid proxy for how downstream brain areas might linearly “read out” information (Diedrichsen & Kriegeskorte, 2017; Kriegeskorte et al., 2008), compared with angular/geometric distance metrics which focus on the overall pattern shape (Kriegeskorte et al., 2008). 2. We also tested correlation-based measures (Pearson and Spearman), but decoding gave stronger effects compared with the correlation measures we tested, allowing us to fuse the modalities with stronger patterns. We converted the decoding accuracies to a dissimilarity metric by calculating the distance from perfect performance: Dissimilarity = 1 – Decoding Accuracy (where accuracy is scaled 0 to 1). We obtained fMRI neural RDMs for every ROI and then averaged them across hemispheres. We also obtained MEG neural RDMs for every time point across the trial, once for the *stimulus-aligned* data and once for *response-aligned* data. MEG RDMs were constructed using signals from all 160 sensors over the whole brain.

We also constructed four theoretical model RDMs to quantify *across-hemifield* and *within-hemifield* stimulus, rule, and response information within neural RDMs explained above (Fig. 2A). For example, in the *across-hemifield stimulus information* model RDM, the elements which corresponded to the decoding of right stimuli from right stimuli, or left stimuli from left stimuli representations (and not their cross conditions) were valued as 0.5, and the elements which corresponded to the cross-conditions between right and left stimuli were valued as 1. For RSA and fusion analyses (explained below), we selected and reshaped the lower triangular elements of the RDMs (excluding the diagonal elements) into vector RDMs (representational dissimilarity vectors: RDVs).

The core of our fMRI–MEG fusion analysis is the Commonality Index, which quantifies the variance shared between three elements: the fMRI RDM (from a specific ROI), the MEG RDM (at a specific time point), and a Template Model RDM (representing a specific task feature, like “Rule”). We use Commonality Analysis (based on multiple regression) to isolate the variance that is uniquely common to all three RDMs. This approach differs from standard partial correlation. The Commonality Index, CI_fMRI,MEG,Model_, directly measures the size of the three-way intersection of the variance explained by the three RDMs (Fig. 2B). This Index ensures that we only extract spatiotemporal dynamics that satisfy a triple constraint:

1. The information is encoded in the fMRI ROI (spatial constraint).
2. The information is encoded in the MEG sensor data at that time point (temporal constraint).
3. The information matches the structure of the task feature (Model constraint).

The use of this three-way metric is highly conservative and necessary to filter out noise unique to a single modality (e.g., haemodynamic noise in fMRI, head motion noise in MEG), providing a robust, cross-modal signature of task-relevant information. Crucially, the Index does not partial out the template–RSA model variance; rather, it measures the shared variance that is explained by the model.

To that end, we obtained one commonality index for each ROI in fMRI at each time point of MEG and each of the four models as defined in (1):

$$Commonality\;(RoI,t,m)=\rho_{fMRI(RoI)MEG(t)}-\frac{\rho_{fMRI(RoI)MEG(t)}-\rho_{fMRI(RoI)Model(m)}\cdot \rho_{MEG(t)Model(m)}}{\sqrt{1-{\left(\rho_{fMRI(RoI)Model(m)}\right)}^{2}\sqrt{1-{\left(\rho_{MEG(t)Model(m)}\right)}^{2}}}},$$ (1)

where $\rho_{fMRI\left(RoI\right)MEG\left(t\right)}$ refers to the *Spearman’s* correlation between the neural fMRI RDV in region RoI and neural MEG RDV at time t (highlighted on the left Venn diagram in Fig. 2B), $\rho_{fMRI\left(RoI\right)Model\left(m\right)}$ refers to the neural fMRI RDV in region RoI and model RDV m, and $\rho_{MEG\left(t\right)Model\left(m\right)}$ refers to the neural MEG RDV at time t and model RDV m. The fraction term is, therefore, the correlation between the fMRI and MEG RDVs where model RDV is partialled out (highlighted on the middle Venn diagram in Fig. 2B). The difference between the two terms (Eq. 1) gives the three-way commonality (highlighted on the right Venn diagram in Fig. 2B). Using this formula we obtained four commonality indices (for the four models) in each ROI at every 5-ms time point.

The Commonality Index, derived from the three-way intersection of fMRI, MEG, and the model, should be interpreted as the robust spatiotemporal signature of task-relevant information. It indicates when a particular task feature (as defined by the models) is represented with the highest reliability across modalities. However, the index does not generate new data or time points. It can only be significant where an effect is already present in the fMRI, the MEG, and the model (or strongly correlated with all three). The rationale for using this index is its powerful ability to filter out noise that is unique to a single modality (e.g., haemodynamic noise in fMRI, head motion noise in MEG), thereby focusing the interpretation on the most reliable, cross-modal neural substrate of the task feature.

### 2.9 Statistical analyses

#### 2.9.1 Bayes factor analysis

We used Bayes factor t-tests, as implemented by Krekelberg^2^ based on Rouder et al. (2012), for comparing the levels of decoding across conditions and between a condition and chance decoding. We used standard rules of thumb for interpreting levels of evidence (Dienes, 2014; Lee & Wagenmakers, 2005): Bayes factors of >3 and <1/3 were interpreted as evidence for the alternative and null hypotheses, respectively. We interpreted Bayes factors between 3 and 1/3 as insufficient evidence either way, but to avoid arbitrary thresholding, we mention whether they are in the direction of the alternative or the null where appropriate and give all BF values to show the strength of evidence.

To evaluate the evidence for the null and alternative hypotheses of at-chance and above-chance decoding in *fMRI*, respectively, we generated a null distribution containing 1,000 decoding values obtained by randomising class labels 1,000 times (random permutation). As we were also interested in evaluating the effect of perceptual difficulty on information coding, we also used Bayes factor analysis to evaluate the evidence for difference in decoding levels between *across-* and *within*-stimulus information across participants in each ROI separately.

To evaluate the evidence for the null and alternative hypotheses of at-chance and above-chance decoding in *MEG*, respectively, we generated a null distribution containing 1,000 decoding values obtained by randomising class labels 1,000 times (random permutation) for every time point. As in fMRI, to evaluate the evidence for difference in decoding levels between *across-* and *within*-stimulus information, we compared their decoding rates across participants at every time point. Accordingly, we performed the Bayes factor analysis for alternative (i.e., difference; H1) versus the null (i.e., no difference; H0) hypotheses.

The priors for all Bayes factor analyses were determined based on Jeffrey–Zellner–Siow priors (Jeffreys, 1961; Zellner & Siow, 1980) from the Cauchy distribution based on the effect size that is initially calculated in the algorithm using a t-test (Rouder et al., 2012). The priors are data driven and have been shown to be invariant with respect to linear transformations of measurement units (Rouder et al., 2012), which reduces the chance of being biased towards the null or alternative hypotheses. We did not perform correction for multiple comparisons when using Bayes factors as they are much more conservative than frequentist analysis (Gelman & Tuerlinckx, 2000; Gelman et al., 2012). However, we placed less weight on sporadic high BFs than on clustered BFs where there is sustained evidence for a difference (or for the null).

#### 2.9.2 Permutation testing for fusion

To evaluate the significance of commonality indices obtained using partial correlation, we tested the true partial correlations against a null distribution obtained by shuffling the class labels in fMRI data and regenerating the RDMs using the data with shuffled labels 1,000 times. As our hypothesis explicitly sought to identify the presence of shared, task-relevant information (i.e., a positive correlation), and since negative commonalities are difficult to interpret, we used a one-tailed test for significance. Specifically, we compared the true commonality indices at every time point with the randomly generated commonality indices for the same time point and deemed it significant if it exceeded 95% of the random correlations (one-tailed p < 0.05) in positive direction after correcting for multiple comparisons across time (using Matlab mafdr function which uses the direct approach, where the algorithm fixes the rejection region and then estimates its corresponding error rate resulting in increased accuracy and power). We used the Storey’s method based on the expected sparsity of our neurophysiological effects. Specifically, we expected that the effects of information coding are sparse and temporally clustered, meaning only a relatively small fraction of time points will show a true effect. Under this common condition where h0 is significantly less than 1, adaptive procedures such as Storey’s are proven to provide a substantial gain in power over the non-adaptive Benjamini–Hochberg method (Yin et al., 2009), ensuring we can detect the actual dynamics of neural representations while still maintaining appropriate False Discovery Rate control (Storey & Tibshirani, 2003). We used a permutation approach rather than Bayes factor analysis here as there was no chance level of commonality known *a priori*. For quantitative comparison of commonality indices, we extracted three parameters from the commonality time courses consisting of “time to first significant commonality” (time from stimulus onset to the first significant commonality), “time to maximum commonality” (time from stimulus onset to the maximum commonality), and “time from maximum commonality” (time from the maximum commonality to the response).

## 3 Results

Participants exhibited relatively high performance (accuracy: mean = 80.78%, sd = 9.29%; correct reaction time: mean = 1,692 ms, sd = 284 ms), indicating that they had learned the task and could successfully implement the rules, despite the task complexity. Participants failed to respond on an average of 2.6% (sd = 3.04%) of trials.

### 3.1 Multivariate pattern analysis in space and time

**Fig. 3.**
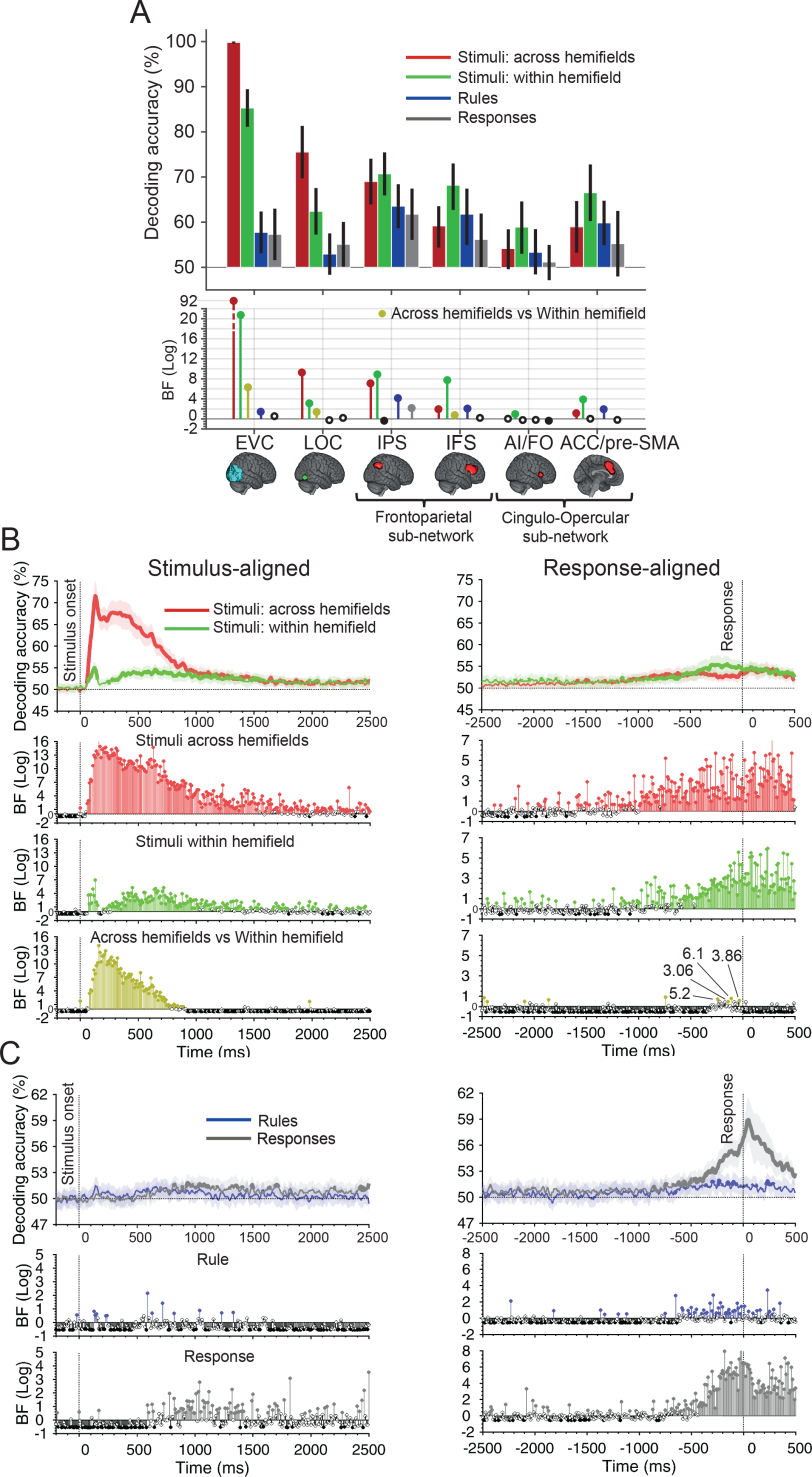
Decoding of different types of information from fMRI ROIs and MEG time points. (A) Average fMRI decoding (error bars = 95% confidence interval across participants) for every ROI. The theoretical chance-level decoding is 50%. Bottom panel: Bayesian evidence for above-chance decoding for each condition (relative to chance) and difference between *across-* and *within*-hemifield stimulus information decoding (yellow), BF: Filled coloured circles show evidence (BF_10_ > 3) for the alternative hypothesis, and filled black circles reflect evidence for the null hypothesis (BF_10_ < 1/3). Empty circles indicate insufficient evidence (1/3< BF_10_ < 3). BFs were calculated using Bayesian t-tests. (B) Stimulus- and response-aligned decoding of stimulus information across time in MEG data. Thick lines show the average across participants (shading 95% confidence intervals), double-thickened lines show time points with evidence (BF_10_ > 3) for above-chance decoding. BFs are shown every 10 ms (rather than 5 ms) for clearer illustration. Horizontal dotted line refers to theoretical chance-level decoding (50%). Vertical dotted lines indicate critical times in the trial. Bottom panels should be read as in (A). (C) Same as (B) for rule and response information. Within-stimulus information, rule and responses were re-analysed from Robinson et al. (2022). Note the different scales of the BF panels.

As an initial step, we evaluated the coding of different types of information using multivariate pattern analysis (MVPA). For this, we used Region of Interest (ROI)-specific fMRI decoding (Fig. 3A) in early visual cortices, the lateral occipital complex (LOC), and the regions that form the MDN.

We quantified the strength of representations about four orthogonal aspects of information using decoding (see Section 2 and Fig. 1A), namely *across*- and *within*-hemifield stimulus information, rule information, and response information. Specifically, for stimulus information, we looked at two possible levels of required precision in representations of stimulus location. To quantify *across-*hemifield (called *across* for brevity) stimulus information, we decoded information about the visual hemifield where the stimulus was presented. To quantify *within*-hemifield (called *within* for brevity) stimulus information, we decoded information about the precise location of the stimulus within a visual hemifield. We compared *across-* and *within*-stimulus information, to test whether these different aspects of the visual representation were encoded in a similar or different manner across the brain, following previous suggestions that the MDN may prioritise difficult (i.e., *within*) over easy (i.e., *across*) aspects of stimulus information (Woolgar et al., 2011a, 2015b). For rule information, we decoded the information about the rule (Rule 1 or 2). For response information, we decoded trials where inner versus outer fingers were pressed.

### 3.2 ROI-based fMRI decoding

In visual cortex, we observed strong representation of stimulus information (Fig. 3A). The classifier was able to distinguish both the broad *across* hemifield (red bars) and fine-grained *within-*hemifield (green bars) stimulus information (*across*-stimulus information BF_10_ for EVC = 1.4 × 10^92^, LOC = 1.6 × 10^9^ and *within*-stimulus information BF_10_ for EVC = 4.6 × 10^20^, LOC = 1.1 × 10^3^), and there was evidence that coding of *across-*stimulus information was stronger than that of *within*-stimulus information (BF_10_ = 1.7 × 10^6^), reflecting the magnitude of the visual differences in *across*-and *within*-stimulus comparisons. There was also evidence that the EVC represented rule information (BF_10_ = 23) but there was borderline evidence in the direction of the null for rule representation in the LOC (BF_10_ = 0.48), and in the direction of the alternative regarding response information representation in the EVC (BF_10_ = 3), with insufficient evidence either way for the LOC (BF_10_ = 1.4).

The MDN, however, exhibited a different pattern of information coding. There was evidence for coding of *across-*hemifield stimulus information in all ROIs except for the AI/FO (BF_10_ for IPS = 1.1 × 10^7^, IFS = 75, ACC = 12, AI/FO = 0.92) and evidence for *within*-stimulus information for all MDN ROIs (BF_10_ for IPS = 6.6 × 10^8^, AI/FO = 7.8, IFS = 46 × 10^6^, ACC = 6.6 × 10^3^). The pattern for stimulus information was opposite to the EVC, with coding of *within-*stimulus information being numerically stronger than coding of *across* information in all regions, and statistical evidence for a difference in the IFS (BF_10_ = 5). There was evidence that rules were represented across all the MDN (BF_10_ for IPS = 1.2 × 10^4^, IFS = 99, ACC = 74) except for the AI/FO (insufficient evidence in the direction of the null; BF_10_ = 0.55). Only the IPS held reliable information about response (BF_10_ for IPS = 130, AI/FO = 0.31, IFS = 1.4, ACC = 0.55).

### 3.3 Time-resolved MEG decoding

In MEG, we used data from a previous study using the same paradigm in an independent group of participants (Robinson et al., 2022). As in that study, we used the stimulus-locked and response-locked analyses to examine different aspects of information processing: whereas stimulus-driven signals are likely to be strongest when locked to stimulus onset, rule application and decision making require computation based on the cue and can take variable amounts of time on different trials, and so is likely to be more evident when we align the neural signals based on the time of response. Here we examine coding of a new aspect of the task (*across*-hemifield stimulus information). We also re-ran the time-resolved decoding of fine stimulus, rule, and response coding reported in Robinson et al. (2022). We reproduce the results here for ease of comparison to the *across*-stimulus information and the fMRI decoding results.

*Across-* and *within*-stimulus information was present (BF_10_ > 3) when the signals were aligned to stimulus onset from 55 ms and 65 ms post-stimulus onset, respectively, both peaking at 130 ms (Fig. 3B, left). Both types of visual information were sustained until after 1,000 ms with evidence (BF_10_ > 3) for stronger *across-* than *within*-stimulus information until 800 ms post-stimulus onset. In contrast, when the signals were aligned to response, there was evidence (BF_10_ > 3) for greater coding of *within-* than of *across*-stimulus information only between 300 and 100 ms before the response (Fig. 3B, right).

For rule coding, we reproduce here the results reported in Robinson et al. (2022). The *stimulus-aligned* analysis showed only a few sparse time points with evidence (BF_10_ > 3) for above-chance rule information after the stimulus onset (Fig. 3C, left, blue), but *response-aligned* analyses showed numerous consecutive time points with evidence (BF_10_ > 3) before and around the time of response (from -500 to 500 ms post-response time; Fig. 3C, right, blue).

We also reproduce here the results for response coding reported in Robinson et al. (2022). There was little response information in the *stimulus-aligned* analysis (Fig. 3C, left, grey) but when the data were *response aligned*, there was evidence (BF_10_ > 3) for response information around the time response starting from around -700 ms and lasting until the end of the analysis window (Fig. 3C, right, grey).

Comparing the fMRI and MEG data qualitatively, we observe that the pattern of information coding in the EVC, with stronger coding of *across-* relative to *within*-stimulus information, and relatively weak evidence for coding of rules and response, mirrors the data from earlier time points in the MEG. Conversely, data from several MD regions, reflecting stronger decoding of *within-* than of *across*-stimulus information, and task rules, more closely mirror the MEG data from later time points. Next, we sought to formalise these observations and study the dynamics of information coding in each region using model-based MEG–fMRI fusion.

### 3.4 Spatiotemporal dynamics of information encoding (fMRI–MEG fusion)

We used model-based fusion of fMRI and MEG representations (Hebart et al., 2018; Moerel et al., 2024) to quantify when, and in which ROI, the structure of representational space in the two modalities correlated with each other and with a theoretical model that captured the *across-*, *within-*hemifield stimulus, rule, and response information (Fig. 2). This allowed us to quantify, for each region, the time course with which different aspects of the task were represented.

**Fig. 4.**
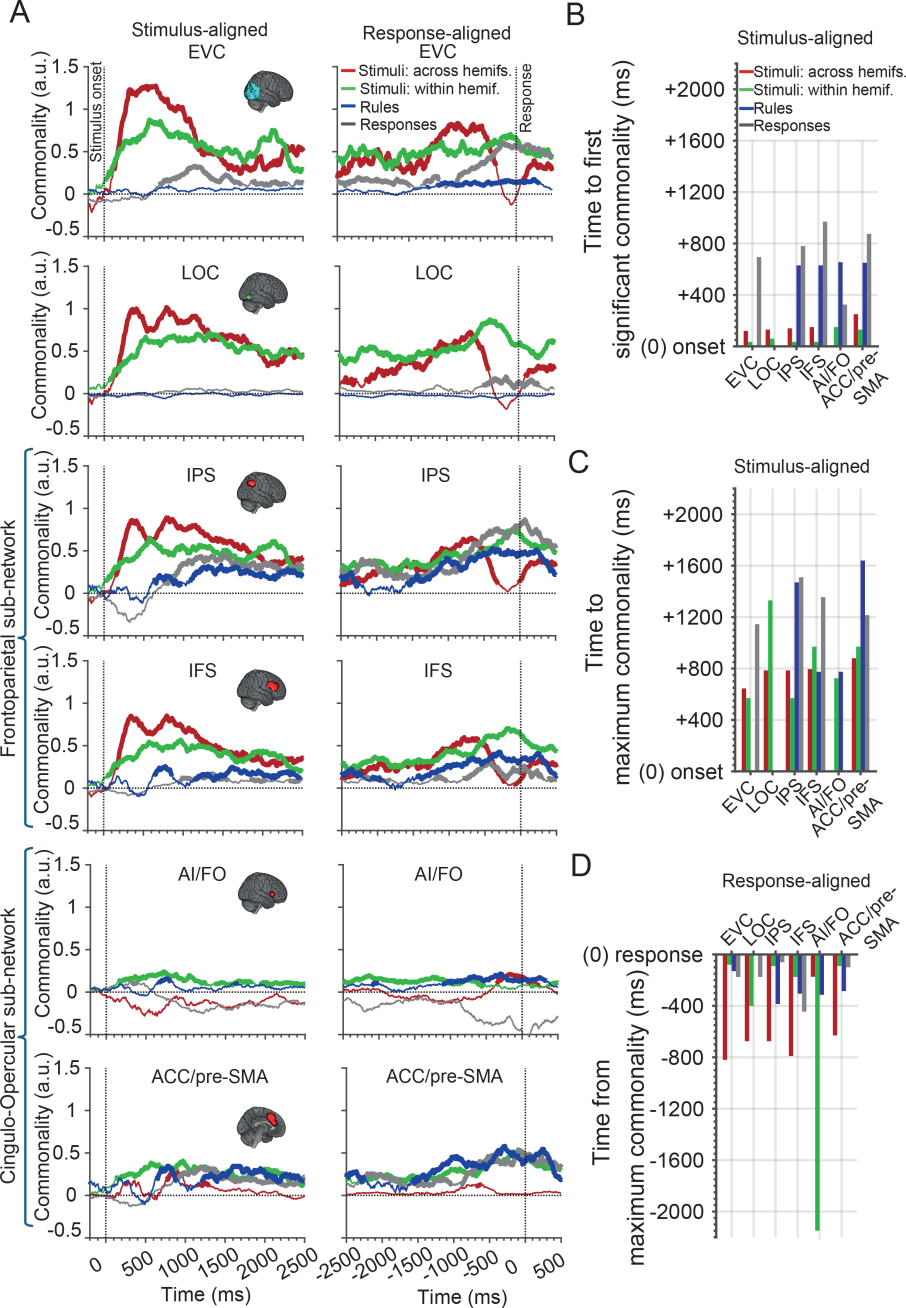
Commonality of information coding between MEG and fMRI. (A) Commonality time courses aligned to the stimulus-onset (left column) and response (right column) times. Thickened lines indicate significant commonalities (p < 0.05; one-tailed random permutation testing; corrected for multiple comparisons across time). Note that only positive commonalities are interpretable as they reflect the correlation between modalities. Horizontal dotted line indicates no commonality, and negative commonalities are not meaningful. Vertical dotted lines indicate critical times in the trial. (B–D) Quantitative commonality metrics: Time from the stimulus onset to the first significant (B) and to maximum (C) commonalities, and the time from the maximum commonality to the response (D), extracted from the commonality time courses for each information type. Bars are only shown for commonality traces that reached significance.

The commonality profiles in general followed the MEG decoding profiles with stimulus information more aligned to the stimulus onset (Fig. 4A, left) and the rule and response commonalities peaking before and around the response (Fig. 4A, right). We used a permutation approach rather than Bayes factor analysis here as there was no way to *a priori* set the chance level of commonality. Significant commonalities (p < 0.05; random permutation testing corrected for multiple comparisons) are shown by thickened lines.

For stimulus information, in the *stimulus-aligned* analysis, there was higher commonality for *across-* than for *within*-stimulus information in EVC, LOC, IPS, and IFS. However, in the *response-aligned* analysis, the commonality was higher for *within-* than for *across*-stimulus information in all these regions around the time of response. Thus, the general pattern seen in the MEG data can be ascribed to the responses of visual and MDN regions, with *across*-stimulus information dominating early and *within*-stimulus information dominating around the time of the response. Intriguingly, if we consider the different nodes of the MDN, there seems to be distinctions, particularly between frontoparietal (IFS, IPS) and cingulo-opercular (AI/FO, ACC) nodes. In the cingulo-opercular sub-network, commonality tended to be weaker and less dynamic (flatter) than the frontoparietal sub-network. Both IPS and IFS showed significant coding of both *within-* and *across*-stimulus information which was dominated by *across*-stimulus information around the time of stimulus representation and dominated by the *within*-stimulus information immediately before the response. By contrast, the AI/FO showed significant coding of the *within* (but not *across*)-stimulus information around the time of stimulus representation, and coding of the *across-* (and not *within-*) stimulus information around the time of the response, at around the time that the frontoparietal sub-network stopped coding the *across*-stimulus information. The ACC/pre-SMA showed a sustained representation of the *within*-stimulus information both after stimulus onset (*stimulus-aligned*) and around the time of the response (*response-aligned*). This may suggest a different role for the AI/FO and ACC/pre-SMA relative to the more lateral and dorsal frontoparietal sub-network of the MDN in stimulus processing, as proposed by previous studies (Crittenden et al., 2016; Dosenbach et al., 2006). These previous studies have found weaker and more sustained activations and coding in ACC/pre-SMA and AI/FO across the task, suggesting they were involved in maintaining task sets, whereas IPS and IFS showed more flexible, transient, and adaptive activation and coding reflecting more variability (Crittenden et al., 2016; Dosenbach et al., 2006).

In the stimulus-aligned analysis, rule information first appeared later in time than the visual information (after ~700 ms) and was seen in all MDN but no visual areas (Fig. 4A, left). In the *response-aligned* analysis, all nodes of the MDN showed the rule information from ~-2,500 ms before the response (ramping up and peaking at ~-500 ms before the response), the EVC showed rule information only appearing later, ~-1,000 ms before the response (Fig. 4A, right). Therefore, the significant rule coding in the EVC in the fMRI data is likely to have arisen from these later time points.

Not surprisingly, the rising temporal profile of response information was more clearly observed in *response-aligned* analysis, which peaked around the time of response in all areas except AI/FO, which did not show response coding.

The commonality results suggest distinct temporal profiles of information encoding between the different types of information and brain regions. To quantify the comparison between different types of information, we calculated “time to first significant commonality,” “time to maximum commonality,” and “time from maximum commonality” for each region and information aspect (see Section 2 and Fig. 4B–D). These are single data values as fusion was performed using group-averaged data across modalities. In *stimulus-aligned* data, the time to the first significant commonality was slightly shorter for the *within-* (green bars) than for the *across-* (red bars) stimulus information in all areas (except for AI/FO where *across*-stimulus information was never significant so we do not have a data point; Fig. 4B). However, the times to maximum commonality for the two types of stimulus information were similar (for all regions except LOC where *across* information was first; Fig. 4C). In *response-aligned* data, commonalities also reached their peaks earlier for *across-*relative to *within*-stimulus information, across all areas except AI/FO, which, as noted above showed a low but sustained representation of the *within* stimulus throughout the analysis window (Fig. 4D).

**Stimulus information:** Comparing regions, relative to the stimulus onset, the time to the *first* commonality for the *across*-stimulus information was numerically shorter for the cingulo-opercular relative to the frontoparietal sub-network of the MDN (Fig. 4B, green bars: IPS = 40 ms; IFS = 40 ms; vs. AI/FO = 150 ms; ACC/pre-SMA = 135 ms), supporting their difference in temporal dynamics. Moreover, the time to the *maximum* commonality increased from posterior visual areas to anterior nodes of the MDN for the *across-* (Fig. 4C, red bars: EVC = 645 ms; LOC = 785 ms; IPS = 785 ms; AI/FO = N/A; IFS = 795 ms; ACC/pre-SMA = 880 ms) and similarly for *within-* (Fig. 4C, green bars: EVC = 570 ms; IPS = 570 ms; AI/FO = 725; IFS = 970 ms; ACC/pre-SMA = 970 ms; except LOC = 1,330 ms) stimulus information. This suggests potential feed-forward flow of stimulus information from EVC to MDN (not including LOC, which the time course suggests it might have been by-passed for *within*-hemifield stimuli).

**Rule information:** In *response-aligned* data, the time from maximum commonality (to response) for rule information suggests that, relative to response times, rule information was dominantly encoded by the posterior MDN areas followed by more anterior areas, and finally appeared in the EVC just prior to the response being actually made (Fig. 4D, blue bars: IPS = -385 ms; AI/FO = -305; IFS = -305 ms; ACC/pre-SMA = -285 ms; EVC = -130 ms; LOC = N/A).

**Response information:** The time from maximum commonality to response for response information in the *response-aligned* data did not show a clear pattern but was shortest in the IPS area (Fig. 4D, grey bars: AI/FO = 1,425 ms; IFS = 445 ms; EVC = 175 ms; LOC = 175; ACC/pre-SMA = 100 ms; IPS = 60 ms), suggesting that this area might be one of the critical ROIs in the MDN to support motor responses (IPS was the only area with significant response information in fMRI c.f., Fig. 3A).

Together, commonality analyses showed that sub-regions of the MDN had distinct temporal dynamics of information coding within the same trial. The fusion-based analysis allowed us to characterise the content and potential direction of the transferred information across the visual and MDN. For example, the temporal dynamics of the commonality traces showed a clear change in the magnitude of information coding in *across*- versus *within*-hemifield stimulus information later in the trial which reflected the dynamical changes within each trial across the MDN. Moreover, we observed later appearance of rule information in EVC than MDN consistent with feedback mechanisms from the MDN to EVC (Fig. 4D). Finally, we saw distinct temporal patterns of information coding across the AI/FO and ACC/pre-SMA relative to the more lateral and dorsal frontoparietal sub-network of the MDN, which aligns with previous studies suggesting close functional cooperation within the cingulo-opercular sub-network and their potential distinction from the frontoparietal sub-network of the MDN (Crittenden et al., 2016).

## 4 Discussion

The multiple demand network has been suggested to be a potential candidate for coordinating distant and functionally distinct areas of the brain to construct “integrated intelligence from distributed brain activity” (Duncan et al., 2020, p.838). However, the temporal dynamics of information coding across the distinct nodes of the MDN have been challenging to investigate, especially in humans. Here we used MVPA in spatially (fMRI) and temporally (MEG) high-resolution neuroimaging modalities and showed that different parts of the MDN represent distinct types of information to varying degrees with subtly different temporal dynamics. We found that coding in the MDN emphasised the more difficult aspects of visual stimuli (i.e., within hemifields), despite this being a smaller physical visual signal, and one that was coded less strongly in visual regions, than large stimulus distinctions (i.e., across hemifields). Then we used the MEG data to show distinct temporal profiles for information about across- and within-hemifield stimuli. Together these results suggested time-varying involvement of MDN and other brain areas in information coding. To formalise this, we fused fMRI and MEG data and showed that there were differences between *across-* and *within*-stimulus information in all areas of the MDN. This provided evidence for dynamic information coding in the MDN that depended on stimulus type and difficulty. We also observed a distinct temporal pattern of information coding across the frontoparietal versus cingulo-opercular sub-networks of the MDN. This provides deeper insights into the distinct roles of different MDN regions in the encoding of multiple types of task information.

Human fMRI studies have shown for two decades that the nodes of the MDN become active (Duncan, 2010; Duncan & Owen, 2000; Fedorenko et al., 2013) and encode (Duncan et al., 2020; Woolgar et al., 2016; Zheng et al., 2024) task-relevant (and usually challenging) aspects of information across a variety of tasks. Due to the temporal resolution of fMRI, however, researchers have been unable to determine whether this apparently simultaneous activation pattern was due to the nodes of the MDN having similar temporal profiles (and, therefore, presumably doing similar things), or a lack of temporal resolution. Here, we combined fMRI with MEG and found detectable differences between the temporal dynamics of information encoding across the nodes of the MDN for distinct aspects of information.

Our analysis provides a detailed, time-resolved account of how distinct task components—stimulus (within and across hemifield), rule, and response—are handled across the visual hierarchy and the MDN. We observed that the earliest information encoding, characterised by across-hemifield stimulus features, was initially confined to the Early Visual Cortex, followed quickly by the Lateral Occipital Complex, aligning with the known feed-forward sweep of visual processing. MDN regions began encoding information shortly after the Lateral Occipital Complex. This detailed picture of the sequence of information processing across the visual and MDN is consistent with the MDN network playing a role in brain-wide information exchange through information integration and propagation (Duncan et al., 2020). Our findings align with an fMRI study that found sensory-related activations (although this study did not look at information) were fed into adjacent nodes of the MDN (Assem et al., 2021). Another recent study found stimulus category information in parietal and frontal brain areas near the core MDN, suggesting potential flow through the MDN (Shashidhara et al., 2024). Together, the additional temporal information that this study provides forms a picture about the way in which the nodes of the MDN and sensory cortices might interact to support goal-driven behaviour.

### 4.1 Segregation of task components across the MDN

We found that, as opposed to visual areas where the coarse *across*-hemifield stimulus information was prioritised over the fine *within*-hemifield stimulus information, there was evidence for the opposite effect in the MDN. We saw (especially in IFS; Fig. 3A) the prioritisation of *within*- over *across-*hemifield stimulus information. Fusion analysis revealed that this prioritisation occurred close to the response time in visual and frontoparietal regions, and throughout the trial in the cingulo-opercular MDN sub-network (Fig. 4A). This suggests that the MDN (especially its cingulo-opercular sub-network) does not passively receive all incoming sensory information; rather, it tunes into task-relevant details that require high-level discrimination, consistent with its hypothesised role as an adaptive coder (e.g., Duncan & Owen, 2000; Woolgar et al., 2011a). We also observed evidence for rule and response information coding in the MDN supporting its role in high-level control mechanisms essential for task performance (e.g., Duncan et al., 2020).

We have previously shown a relationship between stimulus and rule difficulty and the strength of information coding in the MDN (Woolgar et al., 2011a, 2015a, 2015b, 2023). The current results extend previous observations and highlight the role of the MDN in coding the difficult aspects of stimuli without explicit cueing in the experimental design. Specifically, our previous studies compared distinct sets of stimuli (with vs. without noise, e.g., Woolgar et al., 2015b, or at different eccentricities, e.g., Woolgar et al., 2011a) in the difficult versus easy conditions to show the preference of the MDN in coding of difficult versus easy stimulus conditions. As the two stimulus sets differed (in terms of noise or eccentricities), their comparison might have been affected by stimulus-related differences rather than difficulty only.

The tasks also had a blocked design with each block containing either the easy or difficult condition. Therefore, it might have been the case that the higher encoding in the MDN in difficult versus easy blocks reflected a difference in general effort. In the current study, however, we used four stimuli in an event-related design and quantified the level of difficult versus easy aspect of the same stimuli (i.e., *across* hemifields vs. *within* hemifield) in an orthogonal decoding fashion. Specifically, all four stimuli were included in the decoding whether decoding the *across* (left vs. right side) or *within* (inner vs. outer) aspect of the stimulus. Participants needed to process both types of stimulus information on every trial for every single stimulus to successfully perform the task, but because of the proximity of the two stimuli within hemifield, it should be more difficult to discriminate the stimuli within a hemifield compared with the locations across the hemifield (e.g., if a stimulus appeared in the inner left location, it would be easy to discount the two right locations, but harder to determine whether the inner left or outer left was shown). Therefore, the easy versus difficult aspect of stimulus discrimination existed implicitly on every trial rather than being cued. Our results showed that the MDN seems to automatically (without any explicit cues about the difficulty of the information) provide an enhanced representation for the more fine-grained aspect of the visual input, which was not as strongly represented in the visual system as the coarse location information. This is consistent with the role of the MDN in processing difficult aspects of the task (Woolgar et al., 2011a, 2015b). Future studies are needed to assess the generalisability of this preference for more challenging over easier aspects of stimulus encoding by the MDN across other task dimensions (e.g., rule and response).

### 4.2 Functional and temporal segregation within the MDN sub-networks

A primary and novel finding of our spatiotemporal analysis is the evidence for temporal segregation between the MDN sub-networks, providing support for a long-proposed functional dichotomy (Dosenbach et al., 2008). That framework predicts that the cingulo-opercular sub-network would preferentially encode sustained, abstract task elements, whereas the frontoparietal sub-network would handle more transient, episodic task elements. Our fusion analysis supported this interpretation: our results demonstrated distinct temporal dynamics between the cingulo-opercular and frontoparietal sub-networks during stimulus processing. While the frontoparietal sub-network exhibited clearer and more dynamic coding—transitioning from *across*-stimulus dominance to *within*-stimulus information dominance near the response in stimulus-aligned data—the cingulo-opercular sub-network showed more sustained, flatter patterns. Notably, the frontoparietal nodes reached earlier significance for the difficult *within*-stimulus information compared with the cingulo-opercular nodes. This distinct temporal coding profile suggests that the cingulo-opercular sub-network may serve a tonic maintenance role for task sets, providing a stable, overarching cognitive context, while the frontoparietal sub-network serves a more phasic, executive function role, managing the rapid flow and manipulation of current input and output information.

This distinction is consistent with previous findings, which showed that the frontoparietal sub-network generally exhibits stronger and more trial-wise coding than the cingulo-opercular sub-network (Crittenden et al., 2016) and that cingulo-opercular activity is more sustained throughout blocks of trials (Dosenbach et al., 2006; Visscher et al., 2003). Here, we extend this by demonstrating the different cingulo-opercular and frontoparietal temporal dynamics within single trials. Specifically, the frontoparietal sub-network showed dynamic coding of both stimulus types, dominantly representing across-hemifield information at early time points and switching to a dominance of the more fine-grained within-hemifield information around 500 ms before the response. Intriguingly, this pattern was reversed in the AI/FO node of the cingulo-opercular sub-network, which only showed commonality with the challenging within aspect at early time points, but a short period of across-hemifield stimulus information right before the response.

### 4.3 Methodology characteristics, limitations, and future work

While our separate fMRI and MEG MVPA analyses provide key insights, the fusion metric offers a qualitative advantage in reliability and interpretability. First, it increases confidence by reflecting only effects that survive the constraints of both modalities. Second, it provides spatiotemporal precision by locking MEG-level temporal resolution (ms) to fMRI-level spatial resolution (ROI). Separate analyses cannot achieve this: fMRI’s temporal activity is intrinsically blurred by the haemodynamic response, and MEG channel-based or sensor-space analyses lack fMRI’s anatomical precision. By utilising the Commonality Index, we uniquely obtain a spatiotemporal lens onto task-relevant information coding within a single, integrated framework.

Despite the benefits in using fMRI–MEG fusion for obtaining insights about the spatiotemporal dynamics of information coding, there are also limitations (Cichy & Oliva, 2020; Cichy et al., 2014). First, fusion is only useful when effects are present in both modalities; no additional effect will be generated through fusion. If effects appear in one imaging modality but not the other, they will be diminished or not detected. Second, as weak effects might be further weakened in fusion through imperfect correlation across modalities, fusion is more suited for medium-to-large effects that are clear in both modalities. This is particularly the case here, where the fMRI and MEG data come from separate groups of participants. Thus, our study cannot pick-up subtle or less generalisable effects, but our finding of significant commonality, across distinct groups of participants, reflects large effects with generalisable neural mechanisms.

Our hypothesis-driven ROI-based approach in this work allowed us to balance type I and type II errors and interpretability, resolving the subtle temporal differences between MDN regions and sub-networks within a single trial—which would be unattainable with fMRI or MEG alone. However, our tight focus on the specific question of MDN processes means that we cannot draw conclusions about other areas that might potentially be involved. Exploratory whole-brain analyses would be an interesting target for future work.

Because of previous challenges with temporal and spatial resolution in neural recording modalities, there have not yet been strong predictions about the specific involvement of different MDN nodes in the coding of information in an ordered manner. Here we demonstrated that approaching the problem in a data-driven way allows for new insights, such as a possible distinction between the frontoparietal and cingulo-opercular sub-networks of the MDN. Fusion-based analysis can be used in future studies to track the coding and potential flow of different types of information in different tasks. In the case of the MDN, for example, it would be interesting to investigate how the dynamics of information coding change under different circumstances, such as during memory recall, or when directed selective attention is applied.

Our results show that the cingulo-opercular network exhibits sustained coding while the frontoparietal network shows more dynamic, stimulus-to-response transitions. While these findings align with established theories regarding the functional roles of these sub-networks (Dosenbach et al., 2008), we have aimed to avoid reverse inference—in which cognitive states are inferred based on neural data (Poldrack, 2006)—in our interpretation. Instead, by directly quantifying the temporal dynamics and information content within these regions, our data provide the time-resolved evidence needed to move beyond such inferences. Rather than assuming a role based on the region, we characterise the coding properties of the regions themselves, offering a direct observation of the distinct temporal profiles that support suggestions of functional segregation within the MDN system. Future research using causal manipulations, such as Transcranial Magnetic Stimulation, could further establish the necessity of these specific temporal patterns for task execution.

## 5 Conclusions

In conclusion, this study fused fMRI and MEG to provide new insights into the temporal dynamics of information encoding across and beyond the MDN. We showed that the involvement of the MDN varies across different aspects of the task including higher coding of difficult versus easy aspects of stimulus information. We also found temporal differences in the time course of information encoding across the MDN for distinct information types, suggestive of a distinction between the frontoparietal and cingulo-opercular sub-networks of the MDN which may play different roles in information exchange between the MDN and visual areas. This work provides new insights about the potential role of the MDN as one of the main candidate mechanisms supporting complex task performance and raises questions about the differential roles of MDN regions.

## Data Availability

The ethical approval for this study does not allow us to share raw data openly. For data sharing, Institutional Review Board and Data Use Agreements need to be obtained. Source analysis code and the template regions of interest can be provided upon request without institutional agreement.

## Footnotes

1 Information which is represented more strongly can suppress weaker information when the former is partialled out from the latter in RSA, making it harder to detect the weaker information.

2
https://klabhub.github.io/bayesFactor/
